# Intra-cystic concentrations of albendazole-sulphoxide in human cystic echinococcosis: a systematic review and analysis of individual patient data

**DOI:** 10.1007/s00436-016-5054-x

**Published:** 2016-04-16

**Authors:** Felix Lötsch, Judith Naderer, Tomislava Skuhala, Mirjam Groger, Herbert Auer, Klaus Kaczirek, Fredrik Waneck, Michael Ramharter

**Affiliations:** Division of Infectious Diseases and Tropical Medicine Department of Medicine I, Medical University of Vienna, Vienna, Austria; Centre de Recherches Médicales de Lambaréné, Lambaréné, Gabon; University Hospital for Infectious Diseases Fran Mihaljevic, Zagreb, Croatia; Department of Medical Parasitology, Institute of Specific Prophylaxis and Tropical Medicine, Medical University of Vienna, Vienna, Austria; Department of Surgery, Medical University of Vienna, Vienna, Austria; Department of Biomedical Imaging and Image-guided Therapie, Medical University of Vienna, Vienna, Austria; Institut für Tropenmedizin, Universität Tübingen, Tübingen, Germany

**Keywords:** Albendazole, Albendazole-sulphoxide, Drug concentration, Cystic echinococcosis, *Echinococcus granulosus*, Hydatid disease

## Abstract

Cystic echinococcosis (CE) is a widespread zoonosis caused by the species complex *Echinococcus granulosus*. Albendazole (ABZ)—the first-line anthelminthic drug for medical treatment of CE—is metabolized in vivo to the active derivative ABZ-sulphoxide (ABZ-SO). Target-site ABZ-SO concentrations in the hydatid cyst mediate the anthelminthic effect in CE. Primary outcome of this systematic review of individual patient data was the intra-cystic ABZ-SO concentration stratified by cyst size, location, calcification status and use of praziquantel. Studies reporting intra-cystic ABZ-SO concentrations in humans were identified by a systematic search. A pooled analysis of individual patient data was performed to assess intra-cystic concentrations. Pharmacokinetic data of 121 individual cysts were analysed. There was no correlation between plasma and intra-cystic ABZ-SO concentrations (rho = −0.03, *p* = 0.76). Intra-cystic drug concentrations were also not associated with sex and treatment duration. Use of praziquantel in combination with ABZ was associated with higher plasma (median 540 vs. 240 μg/L; *p* = 0.04) but not intra-cystic ABZ-SO concentrations (median 220 vs. 199 μg/L; *p* = 0.36). Relative drug concentrations in hepatic cysts were higher than in other cysts (0.8 vs. 0.4; *p* = 0.05). Intra-cystic concentrations were higher in calcified than non-calcified cysts (median 897 vs. 245 μg/L; *p* = 0.03). There was a trend towards higher intra-cystic concentrations in smaller sized cysts (*β* = −17.2 μg/L/cm; 95th CI, −35.9 to 1.6; *p* = 0.07). This study demonstrates that mean intra-cystic drug concentrations are similar to plasma concentrations on a population level. However, in individual patients plasma concentrations are not directly predictive for intra-cystic concentrations. The use of booster drugs was not associated with higher intra-cystic ABZ-SO concentrations in this analysis.

**Electronic supplementary material:**

The online version of this article (doi:10.1007/s00436-016-5054-x) contains supplementary material, which is available to authorized users.

## Introduction

Cystic echinococcosis is a parasitic zoonosis caused by the larval stage of tapeworms of the species-complex *Echinococcus granulosus*. Humans serve as aberrant intermediate host. The disease is characterised by an almost worldwide distribution causing significant morbidity and considerable socioeconomic impact in highly endemic regions (Budke et al. 2013, 2006).

Humans infected with *E. granulosus* present with cystic lesions in virtually any organ with liver (60 %) and lungs (20 %) being the most commonly affected. Twenty to forty percent of individuals have multiple cysts or multiple organs involved (McManus et al. 2003; Moro and Schantz 2009). Cysts may vary remarkably in size, but are usually between 1 and 15 cm (Eckert and Deplazes 2004). Cysts may persist, grow progressively, collapse, calcify and degrade spontaneously or rupture and release parasitic material. The stage of the disease is defined by radiologic criteria issued by WHO’s informal working group on echinococcosis (WHO-IWGE) (Brunetti et al. 2010). In this classification, cysts of the stages CE1 and CE2 are classified as active, CE3a and CE3b as transitional and CE4 and CE5 as inactive.

Anti-parasitic treatment with albendazole (ABZ) was introduced into clinical practice almost three decades ago (Davis et al. 1986, 1989) and is still one of the cornerstones of management of cystic echinococcosis. It may be used alone or in combination with surgical or interventional techniques. ABZ is a benzimidazole derivative with poor bioavailability when administered without fatty food. Certain drugs (e.g. praziquantel or cimetidine)—when co-administered with ABZ—were reported to increase serum- and potentially intra-cystic drug concentrations (Wen et al. 1994; Cobo et al. 1998). However, these drugs are not yet routinely recommended as it is unknown whether the administration of a booster drug translates into improved clinical outcome.

ABZ-SO acts mainly on the germinal layer of the cyst and only to a lesser extent on protoscolices (Liu et al. 2015). Cure rates of medical treatment were shown to depend on cyst size and stage. Although it is not known whether higher intra-cystic drug concentrations are associated with improved clinical outcome, the mode of action at the target site strongly supports this hypothesis. Similarly, monitoring of ABZ-SO serum levels is recommended but data are lacking whether serum levels are predictive for intra-cystic drug concentrations. Measurement of intra-cystic target site concentrations is therefore currently the best surrogate pharmacokinetic marker for medical treatment of human echinococcosis.

To improve our knowledge on intra-cystic drug concentrations of ABZ-SO in human cystic echinococcosis and to describe its potential determinants, this systematic review and a pooled analysis of collected data was performed. Primary outcome was the intra-cystic ABZ-SO concentration stratified by cyst size, location, calcification status and use of booster drugs. This review provides a systematic collation of all available evidence on intra-cystic concentrations of ABZ-SO in the treatment of cystic echinococcosis in humans and its determinants.

## Material and methods

### Search strategy

A systematic search strategy identifying all relevant information on intra-cystic ABZ and ABZ-SO concentrations in echinococcosis was conceived. To identify eligible publications, “PubMed” and “Cochrane” databases were searched with the search terms “(albendazole*) AND (echinococc* OR hydatid*)” for references published until August 2014 with all publications being considered before this date. There were no predefined language restrictions and non-English papers were translated for further analysis. Inclusion criteria were: (1) human patients (i.e. no animal studies) (2) reporting of intra-cystic drug concentrations of ABZ or ABZ-SO for individual patients.

### Study selection and data collection

**Fig. 1:**
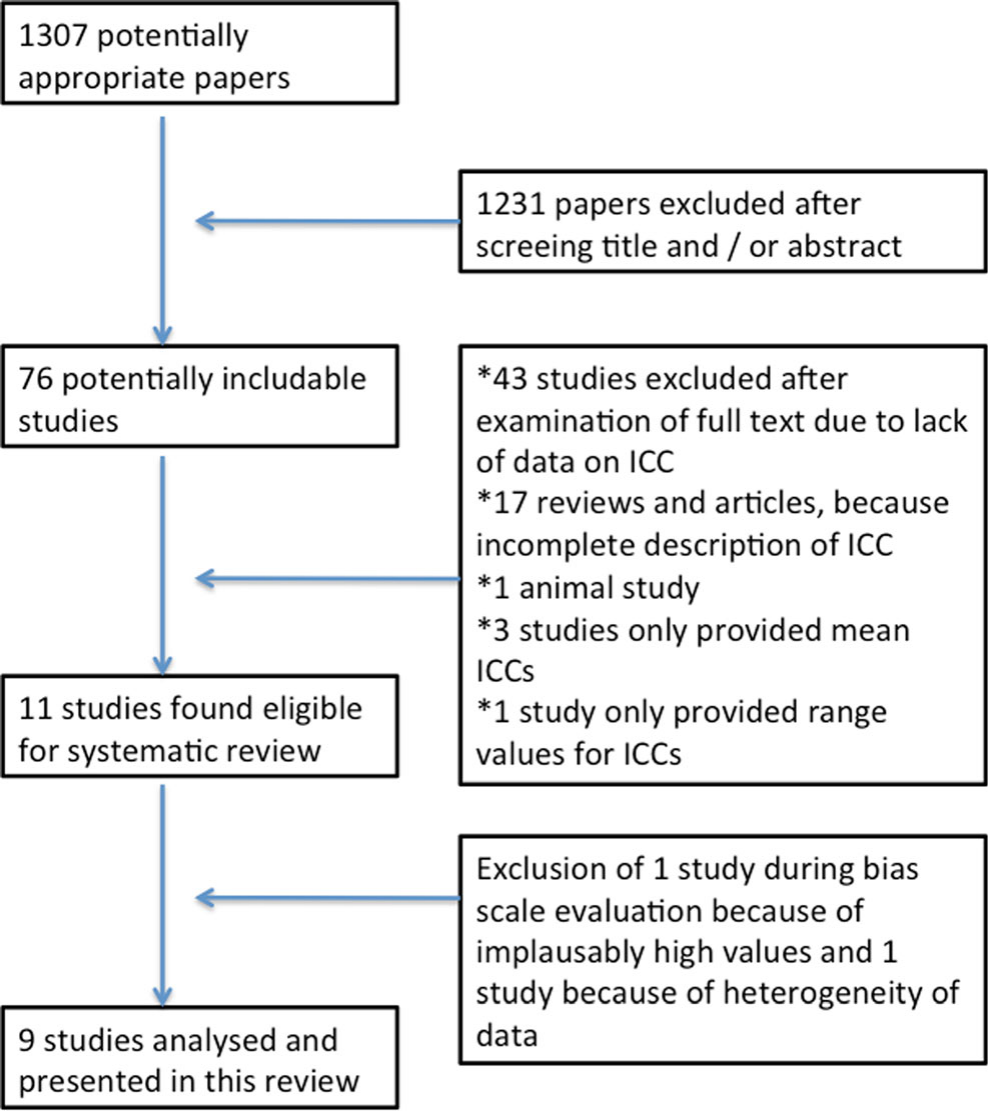
Flow chart showing study selection process

Two independent researchers screened study titles for eligibility. Retrieved references were further assessed for potential inclusion in this review. To assess methodological quality of identified publications, a validation scale was set up and rated by two independent researchers. Evaluation criteria are shown in Supplementary Table 1. Risk for bias was categorized into low, moderate and high following predefined criteria (Supplementary Table 1 and 2). In total, nine studies contributed individual cyst data to the pooled analysis as shown in Table 1. Data were collected and entered into a pre-built database. Data were checked manually by an independent investigator. A flow chart of the study selection process is presented in Fig. 1.

**Table 1:** Summary of included studies with respective variables

Study	Journal	Variables	Contributing cases
Brough et al. (1989)	Aust N Z J Surg	Sex, age, cyst size (*n* = 2), cyst location, calcification status, ABZ-SO intra-cystic concentration, treatment duration, use of praziquantel	3
Capan et al. (2009)	Am J Trop Med Hyg	Sex, age, cyst location, cyst size, ABZ-SO plasma concentration, ABZ-SO intra-cystic concentration, treatment duration, use of praziquantel	2
Cobo et al. (1998)	Trop Med Int Health	Cyst location, ABZ-SO plasma concentration (*n* = 228), ABZ-SO intra-cystic concentration, treatment duration, use of praziquantel	31
Guermouche et al. (1988)	Ann Pharm Fr	ABZ-SO plasma concentration, ABZ-SO intra-cystic concentration, cyst location, treatment duration, use of praziquantel	3
Marriner et al. (1986)	Eur J Clin Pharmacol	ABZ-SO plasma concentration, ABZ-SO intra-cystic concentration, treatment duration, use of praziquantel	4
Morris et al. (1985)	JAMA	ABZ-SO plasma concentration, ABZ-SO intra-cystic concentration, use of praziquantel	3
Morris et al. (1987)	Gut	Sex, age, cyst location, cyst size, ABZ-SO plasma concentration, ABZ-SO intra-cystic concentration, treatment duration, use of praziquantel	18
Saimot et al. (1983)	Lancet	ABZ-SO plasma concentration, ABZ-SO intra-cystic concentration, sex, age, cyst location, calcification status (*n* = 24), treatment duration, use of praziquantel	9
Skuhala et al. (2014)	Croat Med J	Sex, age, cyst location, ABZ-SO plasma concentration, ABZ-SO intra-cystic concentration, treatment duration, use of praziquantel, calcification status	48

Data was available for the following variables: sex (*n* = 80), age (*n* = 68), ABZ-SO plasma concentrations (*n* = 115), ABZ-SO intra-cystic concentrations (*n* = 121), cyst location (*n* = 114), calcification status (*n* = 64), treatment duration (*n* = 118), use of praziquantel (*n* = 121) and cyst size (*n* = 22)

121 cysts in total

### Statistical analyses

An individual patient database was constructed based on the reported data. Each cyst was considered independently in this analysis. Standard descriptive statistics were used to describe the study population. Absolute ABZ-SO concentration in blood and in cyst fluid and relative cyst concentration (blood/cyst fluid) were tested for normal distribution using d’Agostino’s test and visual inspection. Relative drug concentration was defined as intra-cystic drug concentration divided by plasma concentration. Mann–Whitney *U* were used to test differences of drug concentrations for independent samples and Wilcoxon’s rank test for dependent samples. Logistic regression analysis was used to analyse ordinal and metric variables and their influence on drug concentrations. A *p* value of 0.05 or less was considered as statistically significant.

## Results

The systematic literature search identified a total of 1,307 papers, of which 1,231 were excluded by title and abstract. A further 43 studies were excluded due to a lack of data on intra-cystic drug concentrations. Of the remaining studies, incomplete data (*n* = 17), non-human research subjects (*n* = 1) and lack of individual patient data (*n* = 4) led to exclusion of respective reports. One study had to be excluded due to implausibly high values (Glisović et al. 1993) and another study presenting a single case of an intra-cerebral cyst with external shunting (Moskopp and Lotterer 1993) because of the longitudinal design. In the end, data from nine publications were therefore included in this review (Brough et al. 1989; Capan et al. 2009; Cobo et al. 1998; Guermouche et al. 1988; Marriner et al. 1986; Morris et al. 1985, 1987; Saimot et al. 1983; Skuhala et al. 2014) describing drug concentrations in individual hydatid cysts.

### Study population

Altogether, 117 patients were included with a total of 121 analysed cysts. Forty-seven patients were female, 33 were male and in 37 patients sex was not provided. Mean age was 46 years ranging from 6 to 77. Locations of the cystic lesions were as following: liver (*n* = 102), lung (*n* = 3), abdominal wall (*n* = 2), pleura (*n* = 1), spleen (*n* = 1), kidney (*n* = 1), stomach (*n* = 1), retro-peritoneum (*n* = 1), thigh (*n* = 1), bone (*n* = 1) and unknown (*n* = 7). Cyst size was provided in a total of 22 cysts and mean cyst size was 12.2 cm with a range from 4.0 to 30.0 cm. Six cysts were described as calcified, compared to 58 non-calcified cysts. No information on calcification status was provided for 57 cysts.

### Intra-cystic drug concentrations

**Fig. 2:**
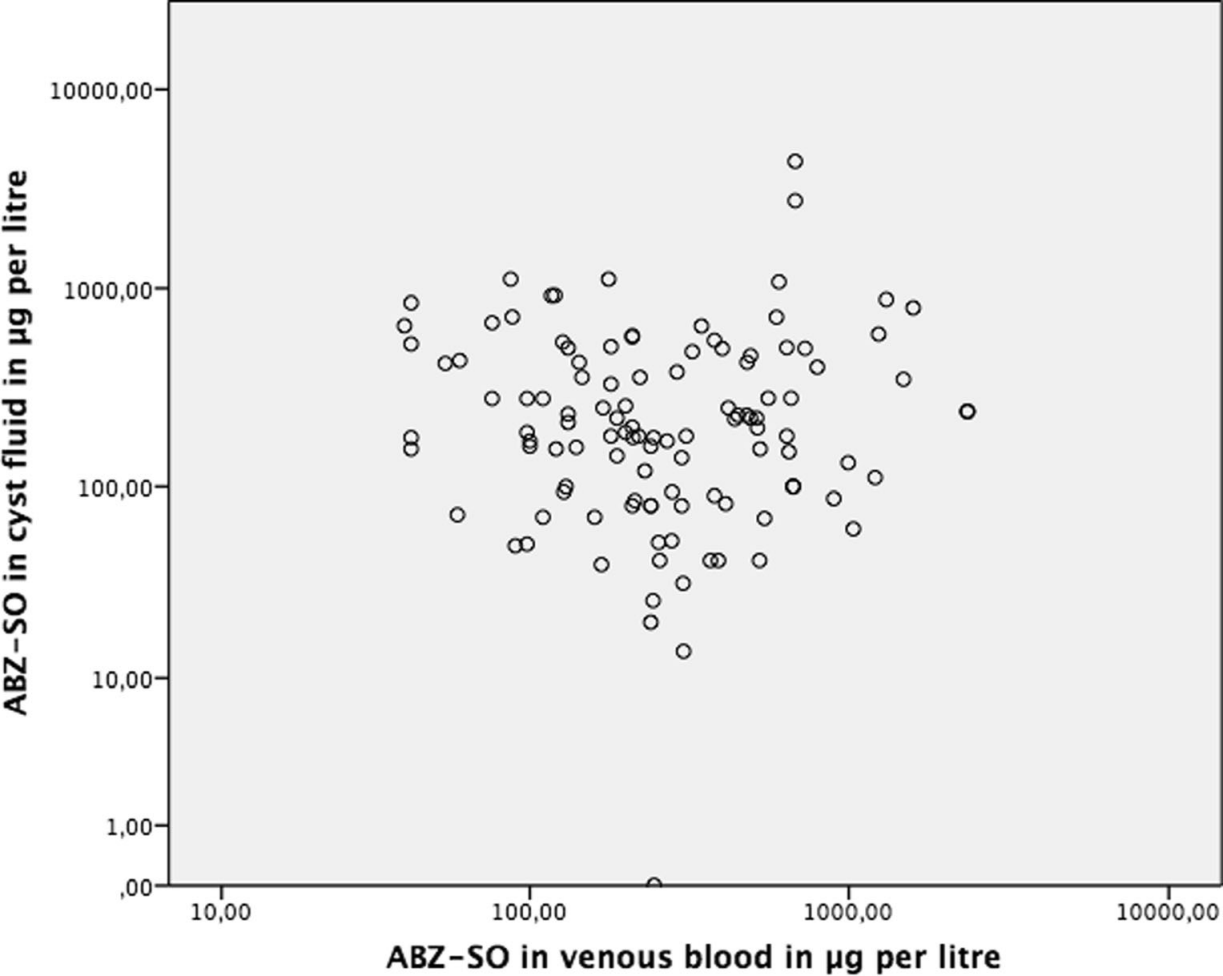
Scatter plot with ABZ-SO in venous blood (plasma) as *x*-axis and intra-cystic ABZ-SO concentration as *y*-axis

The variables ABZ-SO serum, cyst fluid concentrations and relative drug concentrations significantly deviate from a normal distribution (d’Agostino test <0.001 for all variables). Median ABZ-SO concentration in blood was 245 μg/L (25th–75th percentile, 132–518 μg/L) compared to 200 μg/L (25th–75th percentile, 94–434 μg/L) in cysts fluid (*p* = 0.05). Median relative drug concentration was 0.7 (25th–75th percentile, 0.3–1.8). There was no significant correlation between plasma and intra-cystic ABZ-SO concentrations (Spearman, −0.029; *p* = 0.76). A scatter plot of intra-cystic and serum concentrations of ABZ-SO is presented in Fig 2

Comparing blood concentrations of ABZ-SO in patients with cysts in the liver versus other locations, no statistical difference was observed (median 240 μg/L [25th–75th percentile, 132–481 μg/L] vs. 292 μg/L [25th–75th percentile, 140–640 μg/L]; Mann–Whitney *U*, *p* = 0.29). There was also no difference in intra-cystic drug concentrations between liver and non-liver cysts (median 221 μg/L [25th–75th percentile, 111–481 μg/L] vs. 159 μg/L [25th–75th percentile, 82–436 μg/L]; Mann–Whitney *U*, *p* = 0.52). However, relative intra-cystic ABZ-SO drug concentrations were significantly higher in liver cysts compared to non-liver cysts (0.8 [25th–75th percentile, 0.4–2.4] vs. 0.4 [25th–75th percentile, 0.2–0.8]; Mann–Whitney *U*, *p* = 0.05) (Table 2). Median cyst and plasma ABZ-SO concentrations of individual studies are presented in Table 3.

**Table 2:** Table showing plasma, intra-cystic and relative ABZ-SO concentrations in liver vs. non-liver cysts, calcified vs. not calcified cysts, female vs. male patients and in patients with and without praziquantel as booster drug, respectively

	Median (25th–75th percentile)	*p* value (Mann–Whitney *U* test)
Cyst location—liver (*n* = 102) vs. other (*n* = 12)		
Plasma ABZ-SO concentration in μg/L	240 (132–481) vs. 292 (140–640)	0.29
Intra-cystic fluid ABZ-SO concentrations in μg/L	221 (111–481) vs. 159 (82–436)	0.52
Relative cyst concentration	0.8 (0.4–2.4) vs. 0.4 (0.2–0.8)	0.05
Calcification status—calcified (*n* = 6) vs. not calcified (*n* = 58)		
Plasma ABZ-SO concentration in μg/L	640.0 (246–680.0) vs. 218 (114–397)	0.09
Intra-cystic ABZ-SO concentrations in μg/L	897 (504–2,763) vs. 245 (143–499)	0.03
Relative cyst concentration	4.1 (0.8–6.4) vs. 1.2 (0.4–3.3)	0.57
Sex—female (*n* = 47) vs. male (*n* = 33)		
Plasma ABZ-SO concentration in μg/L	200 (121–526) vs. 303 (128–493)	0.39
Intra-cystic ABZ-SO concentrations in μg/L	240 (111–549) vs. 198 (87–425)	0.37
Relative cyst concentration	1.2 (0.2–4.1) vs. 0.7 (0.2–1.6)	0.20
Use of PZQ as booster drug—yes (*n* = 9) vs. no (*n* = 112)		
Plasma ABZ-SO concentration in μg/L	540.0 (255–1,020) vs. 240.0 (132–493)	0.04
Intra-cystic ABZ-SO concentrations in μg/L	220.0 (170–510) vs. 199 (92 vs. 425)	0.36
Relative cyst concentration	0.7 (0.5–0.7) vs. 0.8 (0.2–1.8)	0.81

**Table 3:** Median intra-cystic and venous ABZ-SO concentrations in microgramme per litre of individual studies

	Plasma ABZ-SO concentration in μg/L (25th–75th percentile)	Intra-cystic ABZ-SO concentration in μg/L (25th–75th percentile)
Brough et al. (1989)	n.a.	870 (368–1,080)
Capan et al. (2009)	670 (100–1,240)	375 (160–590)
Cobo et al. (1998)	285 (175–520)	180 (100–280)
Guermouche et al. (1988)	210 (210–210)	570 (200–580)
Marriner et al. (1986)	455 (225–1,510)	90 (50–170)
Morris et al. (1985)	670 (240–2,350)	100 (80–240)
Morris et al. (1987)	304 (240–670)	81 (40–132)
Saimot et al. (1983)	280 (128–640)	504 (158–923)
Skuhala et al. (2014)	200 (104–397)	279 (166–501)

Calcification status was provided for a total of 64 cysts. Six cysts were categorized as calcified compared to 58 non-calcified cysts. Median intra-cystic drug concentrations in calcified and not calcified cysts were 897 μg/L (25th to 75th percentile, 504–2,763 μg/L) and 245 μg/L (25th–75th percentile, 143–499 μg/L), respectively (Mann–Whitney *U* test, *p* = 0.03). Median relative concentrations in calcified and non-calcified cysts were 4.1 (25th–75th percentile, 0.8–6.4) and 1.2 (25th–75th percentile, 0.4–3.3), respectively (*p* = 0.57).

Information on sex of patients was provided for 80 cysts. Forty-seven were female and 33 were male. There was no difference between females and males in blood ABZ-SO concentrations (median 200 μg/L [25th to 75th percentile, 121–526 μg/L] vs. 303 μg/L [25th–75th percentile, 128–493 μg/L]; Mann–Whitney *U* test, *p* = 0.39), cyst fluid concentrations (median 240 μg/L [25th to 75th percentile, 111–549 μg/L] vs. 198 μg/L [25th to 75th percentile, 87–425 μg/L; Mann–Whitney *U* test, *p* = 0.37) and relative drug concentrations (median 1.2 [25th–75th percentile, 0.2–4.1] vs. 0.7 [25th–75th percentile, 0.2–1.6]; Mann–Whitney *U* test, *p* = 0.20) (see Table 2).

In nine cysts, praziquantel was given as a booster drug compared to 112 cysts, in which no praziquantel was administered. The administered dose was 25 mg/kg bodyweight. Mean concentrations of ABZ-SO in blood were 540 μg/L (25th–75th percentile, 255–1,020 μg/L) in the praziquantel group compared to 240 μg/L (25th–75th percentile, 132–493 μg/L) in patients without praziquantel (Mann–Whitney *U* test, *p* = 0.04). Mean concentrations of ABZ-SO in cysts were 220 μg/L (25th–75th percentile, 170–510 μg/L) in the praziquantel group compared to 199 μg/L (25th–75th percentile, 92–425 μg/L) in patients without (Mann–Whitney *U* test, *p* = 0.36). Mean relative cyst concentrations were 0.7 in patients with praziquantel (25th–75th percentile, 0.5–0.7) compared to 0.8 (25th–75th percentile, 0.2–1.8) in patients without (Mann–Whitney *U* test, *p* = 0.79).

Treatment duration before measurement of cyst concentration was provided for 118 cysts. Mean duration was 25 days with a range from 1 to 84 days. In logistic regression, there was no statistically significant influence of treatment duration on ABZ-SO blood concentrations (*β* = 2.1 μg/L; 95th CI, −3.1–7.4; *p* = 0.42), intra-cystic ABZ-SO concentrations (*β* = −5.7 μg/L; 95th CI, −12.2–0.8; *p* = 0.09) and relative concentrations (*β* = 0.00; 95th CI, −0.05–0.04; *p* = 0.89).

Data on cyst size was available for 22 cysts. In linear regression analysis, there was an inverse correlation between cyst size and intra-cystic ABZ-SO concentrations (*β* = −17.2 μg/L; 95th CI, −35.9–1.6; *p* = 0.07), though not reaching statistical significance. No correlation was observed between cyst size and relative drug concentration (*β* = 0.00; 95th CI, −0.04–0.05; *p* = 0.91).

## Discussion

In this systematic review and pooled analysis, data on intra-cystic ABZ-SO in human echinococcosis from all published reports is provided. Intra-cystic drug concentrations were only slightly lower than plasmatic concentrations indicating that ABZ-SO penetrates well into the cyst. At the same time, no intra-patient correlation between blood and intra-cystic ABZ-SO concentrations was observed in this analysis. Individual studies included in this review provided contradictory results. Whereas Skuhala et al. concluded in their study that intra-cystic ABZ-SO concentrations cannot be predicted from plasma concentrations (Skuhala et al. 2014), two other studies found evidence for a correlation between plasma and intra-cystic concentrations (Cobo et al. 1998; Saimot et al. 1983). These findings demonstrate a high inter-personal variability of drug distribution between blood and intra-cystic fluid. Conclusively, ABZ-SO plasma concentrations are not reliable surrogate markers for intra-cystic drug concentrations, which convey the parasitocidal effects.

Intra-cystic ABZ-SO concentrations were also found to be higher in calcified cysts compared to non-calcified lesions. Calcification of hydatid cysts is a sign of a transitional or inactive stage and may lead to a loss of an active barrier function by the parasite against drug penetration and may thus explain a higher influx of the drug. An inverse correlation between cysts size and intra-cystic drug concentration was observed in the pooled analysis. This finding is in line with clinical experience of medical treatment, which is known to be less efficacious in larger sized cysts (Stojkovic et al. 2009). Whether higher dose regimens of ABZ may effectively circumvent this pharmacokinetic issue will ultimately depend on the bioavailability and tolerability of the drug. Further studies are needed to address this issue and to obtain clinical data.

The effect of praziquantel (PZQ), which has been used as a booster drug for ABZ, was evaluated in this analysis and significantly higher blood concentrations of ABZ-SO were demonstrated. However, no difference in intra-cystic and relative concentrations was observed indicating that target site concentrations are not improved by the addition of PZQ. This finding does not speak against a beneficial role of praziquantel in the management of CE, but challenges its use as booster drug to increase intra-cystic concentrations. However, case numbers were limited and further trials would be helpful to further evaluate the benefit of the drug in the setting of human cystic echinococcosis. Further, the lack of association between treatment duration and intra-cystic drug concentrations provides evidence against significant accumulation of ABZ-SO in human hydatid cysts. This might be interpreted in a way that better response rates in long term treatment are conveyed by longer exposure of the parasite to ABZ-SO rather than accumulation of ABZ-SO.

Interestingly, higher relative drug concentrations were found in hepatic cysts compared to cysts of other locations without difference in absolute intra-cystic concentrations. It can be speculated whether the high vascularization and the metabolisation of ABZ to ABZ-SO in the liver are responsible for this effect. Studies of intra-cystic drug concentrations in individual patients with cysts at multiple sites could provide further valuable information.

This systematic review aimed to circumvent the most important limitations in clinical research in the field of echinococcosis, notably small sample size and lack of comparability between sites. A total number of 121 cysts were identified for pooled analysis providing a sufficient sample size to address the main research questions. Based on the systematic literature search, lack of exclusion of foreign language manuscripts, and the distribution of extracted data, we are confident for study publication bias being low. However, due to lack of reported additional information, sub-groups were relatively small leading to inconclusive evidence for some secondary outcomes. These outcomes of this systematic review should therefore be scrutinized in future clinical studies or updates of published evidence. Current international research consortia may serve as an ideal platform for these academic undertakings. Additionally, ABZ-SO concentrations in the intra-cystic fluid were measured although the main site of action is the germinal layer. However, we considered intra-cystic concentrations as the best available surrogate parameter for target site concentrations (Liu et al. 2015).

In summary, this reviews shows that we are lacking good pharmacokinetic data of ABZ and ABZ-SO and its interaction with the cyst in CE. From the few available reports ABZ-SO drug distribution into cysts seems to be satisfactory despite considerable inter-patient variability. The usefulness of measuring plasma concentrations as surrogate parameter for target site concentrations should be tested in a formal trial. Target site concentrations are comparatively higher in small or calcified cysts. Future research should evaluate the clinical impact of these findings and whether pharmacokinetic optimization of therapeutic regimens may improve medical treatment of human cystic echinococcosis.

## Electronic supplementary material

Below is the link to the electronic supplementary material.

ESM 1 (DOC 37 kb)

ESM 2 (DOC 36 kb)
