# Supplementary material for: Intra-cystic concentrations of albendazole-sulphoxide in human cystic echinococcosis: a systematic review and analysis of individual patient data
Source: Parasitol Res. 2016 Apr 16;115:2995–3001. doi: 10.1007/s00436-016-5054-x (PMC4958128; doi:10.1007/s00436-016-5054-x)
Supplement: Supplementary file 1 — (DOC 37 kb) [file 436_2016_5054_MOESM1_ESM.doc]

**SUPPLEMENTARY TABLES**

Supplementary Table 1: Study bias risk evaluation scale

| **1. Detailed dosage and regimen information** | |
| --- | --- |
| *Good* | Exact definition in mg/kg/d or mg/d plus exact treatment duration reported |
| *Sufficient* | Either exact dosage or treatment duration missing, or no exact value reported (as range) |
| *Insufficient* | Neither dosage nor treatment duration reported |
| **2. Data on co-variables (size, location, calcification of the cysts, additional booster drugs, type of hydatid disease)** | |
| *Good* | > 2 of the investigated co-variables reported |
| *Sufficient* | >=1 of the investigated co-variables reported |
| *Insufficient* | None of the investigated co-variables reported |
| **3. Measurement data of plasma and intra-cystic ABZ-SO concentrations:** | |
| *Good* | Data complete, correct units, no rounding errors, realistic values |
| *Sufficient* | Incorrect units and/or data partly complete, or values only shown in a graph, but no rounding errors or incorrect values |
| *Insufficient* | Incomplete data or rounding errors, or unrealistic values and/or incorrect units |
| **4. Demographic data given** | |
| *Good* | Gender, age and weight of patients reported |
| *Sufficient* | At least one item of demographic data reported |
| *Insufficient* | No demographic data reported |
| **5. Time of measurement of target site concentration.** | |
| *Good* | Time of cyst fluid sample extraction defined and within treatment period |
| *Sufficient* | Time of cyst fluid sample extraction within treatment period at least in one patient of the study |
| *Insufficient* | Time of cyst fluid sample extraction outside of treatment period |
