# Supplementary material for: Intra-cystic concentrations of albendazole-sulphoxide in human cystic echinococcosis: a systematic review and analysis of individual patient data
Source: Parasitol Res. 2016 Apr 16;115:2995–3001. doi: 10.1007/s00436-016-5054-x (PMC4958128; doi:10.1007/s00436-016-5054-x)
Supplement: Supplementary file 2 — (DOC 36 kb) [file 436_2016_5054_MOESM2_ESM.doc]

Supplementary Table 1: Bias risk evaluation of included studies

| **Study** | **Dosage regimen** | **Co-variables** | **Measurement data** | **Demographic data** | **Time of measurement** | **Bias risk** |
| --- | --- | --- | --- | --- | --- | --- |
| **Brough**^*^ **1989** | Good | Good | Sufficient | Sufficient | Good | Moderate |
| **Capan 2009** | Good | Good | Good | Sufficient | Good | Low |
| **Cobo 1998** | Good | Sufficient | Sufficient | Insufficient | Good | Moderate |
| **Glisovic 1993** | Good | Good | Insufficient | Sufficient | Good | High |
| **Guermouche**^§^ **1988** | Sufficient | Sufficient | Sufficient | Insufficient | Sufficient | Moderate |
| **Marriner 1986** | Good | Sufficient | Good | Insufficient | Good | Moderate |
| **Morris 1985** | Sufficient | Sufficient | Good | Insufficient | Sufficient | Moderate |
| **Morris 1987** | Good | Good | Good | Good | Good | Low |
| **Saimot 1983** | Sufficient | Good | Sufficient | Sufficient | Good | Moderate |
| **Skuhala 2014** | Good | Good | Sufficient | Sufficient | Good | Low% |

*^*^Note that for the study published in 1989 by Brough et al. the units of one patient were changed from mg/l to μg/l for plausibility.*

*^§^Units presented in table 3 of the paper by Guermouche et al. were changed from mg/ml to ng/ml as mg/ml for plausibility.*

*%Measurement and demographic data were provided by the authors on request.*

*The study of Moskopp et al. was not included due to the heterogeneity of the presented case compared to the other data.*
